# Chronic pain, opioids and addiction

**DOI:** 10.4102/sajpsychiatry.v24i0.1269

**Published:** 2018-10-25

**Authors:** Shaquir Salduker

**Affiliations:** 1 The South African Society of Psychiatrists (SASOP), South Africa

## Abstract

This talk aims to make a distinction between the two growing epidemics in medicine and more especially in psychiatry-opioid dependence and functional pain syndromes (FPS). The distinction has been blurred in current literature, and often, patients with FPS are labelled addicts. Opioid addicts are not treated for the FPS which perpetuate their addiction. More importantly, the onset of both these syndromes is often iatrogenic in nature. Genetics, epigenetics, early childhood trauma and rejection, personality disorders as well as clinical depression and anxiety syndromes all have an intersecting role in this group of patients which makes it crucial for psychiatrists and registrars to be aware of how to recognise and treat them. The Durban pain clinic runs a multidisciplinary team approach towards helping patients with FPS and opioid addictions using the currently available information of the possible pathophysiology of the FPS group of diagnosis, and the basis of this programme will be elaborated and put forward as an alternative to what’s currently available. The clinic with the aid of an unrestricted grant from Cipla, South Africa, is putting together a think tank to come up with guidelines to be published which can be used nationally to manage patients with FPS. The talk proposes these guidelines to the audience and hopes to engender an awareness and level of interest in the field so that the word can be spread amongst other specialties and colleagues. There is no role for opioids in the management of chronic pain syndromes.

